# Modeling and Parameter Identification of a 3D Measurement System Based on Redundant Laser Range Sensors for Industrial Robots

**DOI:** 10.3390/s23041913

**Published:** 2023-02-08

**Authors:** Guanbin Gao, Liulin Kuang, Fei Liu, Yashan Xing, Qinghua Shi

**Affiliations:** 1 Faculty of Mechanical and Electrical Engineering, Kunming University of Science and Technology, Kunming 650500, China; 2 Yunnan International Joint Laboratory of Intelligent Control and Application of Advanced Equipment, Kunming University of Science and Technology, Kunming 650500, China; 3 Yunnan Institute, China Academy of Machinery Science and Technology Group Co., Ltd., Kunming 650031, China

**Keywords:** calibration, identification, laser range sensor, standard spherical constrain, measurement

## Abstract

The low absolute positioning accuracy of industrial robots is one of the bottlenecks preventing industrial robots from precision applications. Kinematic calibration is the main way to improve the absolute positioning accuracy of industrial robots, which greatly relies on three-dimensional (3D) measurement instruments, including laser trackers and pull rope mechanisms. These instruments are costly, and their required intervisibility space is large. In this paper, a precision 3D measurement instrument integrating multiple laser range sensors is designed, which fuses the information of multiple redundant laser range sensors to obtain the coordinates of a 3D position. An identification model of laser beam position and orientation parameters based on redundant distance information and standard spherical constraint is then developed to reduce the requirement for the assembly accuracy of laser range sensors. A hybrid identification algorithm of PSO-LM (particle swarm optimization Levenberg Marquardt) is designed to solve the high-order nonlinear problem of the identification model, where PSO is used for initial value identification, and LM is used for final value identification. Experiments of identification of position and orientation, verifications of the measuring accuracy, and the calibration of industrial robots are conducted, which show the effectiveness of the proposed 3D measurement instrument and identification methods. Moreover, the proposed instrument is small in size and can be used in narrow industrial sites.

## 1. Introduction

The application of industrial robots has continuously enhanced the automation and intelligence of the manufacturing industry. Compared with traditional machine tools, Industrial robots have the advantages of low cost and high flexibility and are increasingly used in grinding, milling, or 3D printing [1]. The requirements for the positioning accuracy of industrial robots are constantly upgrading in many fields [2,3], e.g., aerospace, automobile manufacturing, machining, etc. Generally, the repetitive positioning accuracy of industrial robots can reach the order of 0.01 mm, while the absolute positioning accuracy can only reach the order of 1 mm without calibration [4,5,6]. However, this order of absolute positioning accuracy limits industrial robots in relatively high-precision applications, such as measurement, milling, grinding, etc. The absolute positioning accuracy for an industrial robot is determined not only by its quality of components, manufacture, and assembly but by its mechanical degradation, which is mainly affected by the deformation of linkages, collision, temperature change, and other factors in practice [7,8,9,10]. Kinematic calibration is the major method to improve the absolute positioning accuracy of industrial robots, which includes four steps: establishing a kinematic model, measuring the end position of the robot, identifying the parameters of the kinematic model using the measured data, and compensating in the motion controller with the identified parameters to improve the absolute positioning accuracy of the robot [11]. The influence of thermal drift is important for high-precision industrial robots. After kinematic calibration, the error caused by non-kinematic factors such as thermal drift can be compensated to make the industrial robot obtain higher positioning accuracy [12].

The accuracy of measuring is extremely important for kinematic calibration, which is usually required to be higher than 0.2 mm in the three-dimensional (3D) space. At present, there are few instruments that can meet this requirement in practice, mainly including laser trackers, coordinate measuring machines (CMMs), pull rope calibration mechanisms, etc. [13,14,15]. However, CMMs with large volumes are rarely used for kinematic calibration for industrial robots since the site for kinematic calibration in practice is relatively small.

The laser tracker and the pull rope calibration mechanism are the widely used measuring instruments in robot calibration. For the laser tracker, it is necessary to install one or more reflective targets on the flange of the industrial robot to be measured. The laser emitted by the laser tracker is reflected by the targets to track and measure the end position of the industrial robot in 3D space. Gao et al. [16] measured the end position of an ER20-C10 industrial robot with an API R-20 Radian laser tracker for kinematic calibration, and the maximum positioning errors of the robot along *x*, *y*, and *z* decreased from 3.17 mm, 3.26 mm, and 3.30 mm to 0.39 mm, 0.68 mm, and 0.56 mm after calibration, respectively. Jiang et al. [17] calibrated an RS10N robot using a Leica laser tracker, and the maximum positioning error of the robot decreased from 4.8867 mm to 0.6421 mm. Hsiao et al. [18] completed the calibration of a PMC6VA030 industrial robot with a Faro laser tracker. The maximum positioning error and the average positioning error of the robot decreased from 6.294 mm and 2.613 mm to 1.225 mm and 0.310 mm after calibration.

Different from the laser tracker, the pull rope calibration mechanism uses one end of the rope of the mechanism wound on a photoelectric encoder. The other end is connected to the end flange of the industrial robot through a universal adapter. The displacement of the end of the robot can be measured by the length of the rope pulled out [19]. Li et al. [20] calibrated a 6-DOF robot by using a pull rope calibration mechanism with the result that the maximum positioning error of the robot decreased from 3.36 mm to 1.07 mm. Mei et al. [21] used a pull rope calibration mechanism based on SICK BCG13-E1BM0599 to measure the movement distance of a 4-DoF stacking robot, and the 3σ value of positioning error decreased from 11.73 mm to 1.79 mm after calibration, where σ is the standard deviation.

The above studies show that the calibration results with laser trackers are more accurate than with the pull rope calibration mechanism. However, in practice, the operation of the laser tracker is complex and time-consuming, the required intervisibility space is large, and the cost is also very high [5]. These factors limit its field calibration application in the workshop. Compared with the laser tracker, the measurement accuracy of the pull rope calibration mechanism is much lower, and there is information conversion from distance to position in the calibration model. As a result, its calibration accuracy is limited. Nevertheless, the cost of the pull rope calibration mechanism is also lower than the laser tracker.

Some alternative methods have been presented to meet the increasing demand for calibration in various industries. Boby et al. [22] proposed a calibration method for the industrial robot using a monocular camera as the measuring equipment, and the maximum error of the robot decreased from 8.74 mm to 5.13 mm after calibration. Yang et al. [23] proposed a kinematic calibration method based on the dynamic measurement of double ball linkages. After calibration, the average roundness error (the roundness error is the movement error of a circular path of the robot’s end) of the 6-DOF robot decreased from 0.46 mm to 0.36 mm, and the range of motion errors of the robots’ end in *x* and *y* directions decreased by 0.10 mm and 0.07 mm, respectively. He et al. [24] proposed a kinematic calibration method to improve the accuracy of a TKB2600 industrial robot using multiple location constraints. After calibration, the average relative offset decreased more than 50%. Icli et al. [25] presented an automated calibration method for industrial robots based on three orthogonal plunger dial indicators and four reference balls. After calibration, the maximum position error and average position error of the KUKA KR6 R700 industrial robot decreased to 0.624 mm and 0.326 mm, respectively. Guo et al. [26] presented a 3D measurement system (R-test) with three range sensors and used this equipment to calibrate the HSR-JR605 industrial robot. After calibration, the ratio of distance error to distance and the ratio of relative position error to the relative position error of the robot decreased from 14.30 µm/mm and 20.15 µm/mm to 4.4 µm/mm and 10.85 µm/mm, respectively. The R-test requires the laser beam of the laser range sensor to be strictly orthogonal. The three-dimensional measurement system based on the R-test relies on a high-precision displacement platform in the kinematic calibration of industrial robots. However, the calibration method based on vision measurement is affected greatly by camera distortion, external light, and target accuracy. Moreover, the calibration method based on the dynamic measurement of double ball linkages has a limited measuring range and can only improve the positioning accuracy in the *x* and *y* directions. The calibration method based on external constraints is usually limited by the need to constrain the end of the robot at a point or contact closely with a plane. Thus, the measured information is less, and only zero-point calibration can be performed.

To address the issues of current measurement instruments in the field of robot calibration, we developed a spatial position measurement system (SPMS) based on redundant laser range sensors for measuring the position and calibrating robots in industrial sites. The contributions and innovations of this paper are as follows.

▪ A small precision 3D position measuring device SPMS is proposed, which fuses the information of multiple laser range sensors. The distance information of laser range sensors is converted into position information through the standard spherical constraint to realize the 3D position measurement in a narrow space;
▪ An identification model of laser beam position and orientation parameters based on redundant distance information and standard spherical constraint is proposed to reduce the requirement for the assembly accuracy of laser range sensors;
▪ To solve the high-order nonlinear problem of the identification model, a hybrid identification algorithm is proposed, where PSO is used for initial value identification and LM is used for final value identification;
▪ Experiments were conducted to verify the measuring accuracy of the proposed device.

The paper is structured as follows. Section 2 provides the principle and construction of the novel measurement system. The data acquisition method based on reference sphere constraint, the establishment of the measurement coordinate system, and the identification model of the position and orientation of the laser beam are given in Section 3. A parameter identification method of position and orientation of the laser beam based on a hybrid algorithm of PSO-LM is introduced in Section 4. The experiment of identification of laser beams and verifications of the measuring accuracy and calibration of industrial robots are shown in Section 5. Section 6 concludes this paper.

## 2. Construction of the Measurement System

The measurement system is constructed with redundant laser range sensors to measure a reference sphere installed on the end of the industrial robot and moving along with the robot. The components and measurement principles of the system are elaborated on in this section. The sphere is only mounted on the flange for the robot calibration and not for the later experiments for parameter identification.

### 2.1. Components of the Measurement System

**Figure 1:**
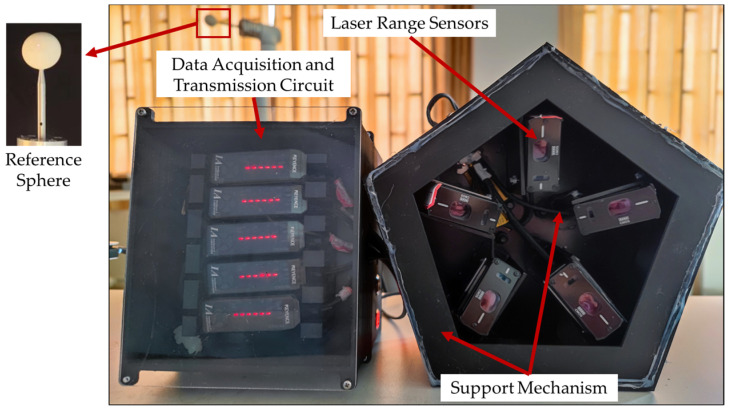
The spatial position measurement system (SPMS).

The proposed SPMS consists of laser range sensors, a reference sphere, a data acquisition and transmission circuit, and a support mechanism. In order to obtain accurate coordinates of the spherical center of the reference sphere, the laser beams in the measuring system should not interfere with each other, and the facula formed by laser beams projected on the reference sphere should be evenly distributed. Therefore, the laser displacement sensors in SPMS are installed in the regular polygon support mechanism, as shown in Figure 1.

**Figure 2:**
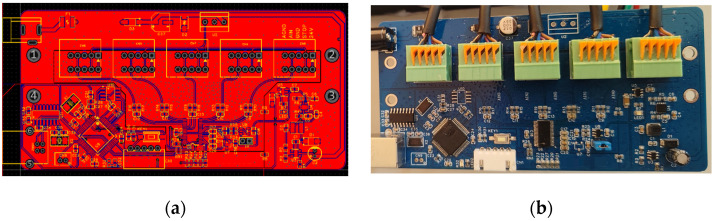
The schematic diagram and photo of the circuit, they should be listed as: (**a**) The schematic diagram; (**b**) the photo of the circuit.

The performance parameters of the range sensor are shown in Table 1. RC (resistor-capacitance circuit) filtering and high-performance ADC (analog-to-digital converter) modules are used to collect, filter and convert the analog signal output by the laser range sensors in the data acquisition and transmission circuit. The digital signal is then transformed into distance information through the MCU (microcontroller unit). The schematic diagram and photo of the circuit are shown in Figure 2.

### 2.2. Measurement Principle of the System

When SPMS is working, the beam of each laser range sensor is projected on the reference sphere at the same time. The coordinates of the flare on the reference sphere are substituted into the spherical equation to obtain the position of the reference sphere center, and then the robot end position is finally measured according to the conversion relationship between the position of the reference sphere center and the position of the robot end.

The coordinates of the flare on the reference sphere can be calculated by:

(1) $$F_{k}=P_{Lk}+\gamma_{k}L_{k}$$

where *k* is the number of laser range sensors (*k* = 1, 2, …, *K*, *K* is the total number of laser range sensors in SPMS); **F***_k_* is the coordinates of the flare; *L_k_* is the length of the beam that can be read from the sensor; and **P***_L__k_* and **γ***_k_* are the position and orientation vectors of the laser beam, respectively, which are determined by the installation of the sensor.

Since **F***_k_* is on the surface of the reference sphere, it conforms to the spherical equation, as shown in (2).

(2) $${\left(x_{k}-x_{B}\right)}^{2}+{\left(y_{k}-y_{B}\right)}^{2}+{\left(z_{k}-z_{B}\right)}^{2}=R^{2}$$

where *x_k_*, *y_k,_* and *z_k_* are the coordinates of **F***_k_* in the *x*, *y,* and *z* direction, respectively; *x_B_*, *y_B,_* and *z_B_* are the coordinates of the center of the reference sphere **P***_B_* in *x*, *y,* and *z* direction, respectively; *R* is the radius of the reference sphere. The coordinates of **P***_B_* are what we want for the reference sphere installed on the end of the robot, which can stand for the position of the robot. The coordinates of **P***_B_* can be written as the function of **P***_L__k_*, **γ***_k_*, *L_k,_* and *R*:

(3) $$P_{B}=f(P_{Lk},\gamma_{k},L_{k},R)$$

where *L_k_* can be read from the sensor; *R* is a known constant; and **P***_L__k_* and **γ***_k_* are unknown constants that can be obtained by precision installation of the sensors or identification.

The reference sphere is fixed at the robots’ end for kinematic calibration. The center position of the reference sphere represents the end position of the robot. That is, **P***_B_* is the end position of the industrial robot.

However, installing the sensor at a certain position and orientation precisely is very difficult, costly, and time-consuming. We proposed an identification method to obtain **P***_L__k_* and **γ***_k_* with redundant laser range information and spherical constrain.

## 3. Data Acquisition and Modeling for Position and Orientation of the Laser Beam

We propose the data acquisition method based on reference sphere constrain. Then, the measurement coordinate system for the SPMS is established based on Schmidt orthogonalization and normalization. Moreover, the position and orientation of the laser beam are modeled with the spherical equation.

### 3.1. Data Acquisition Method Based on Reference Sphere Constraint

**Figure 3:**
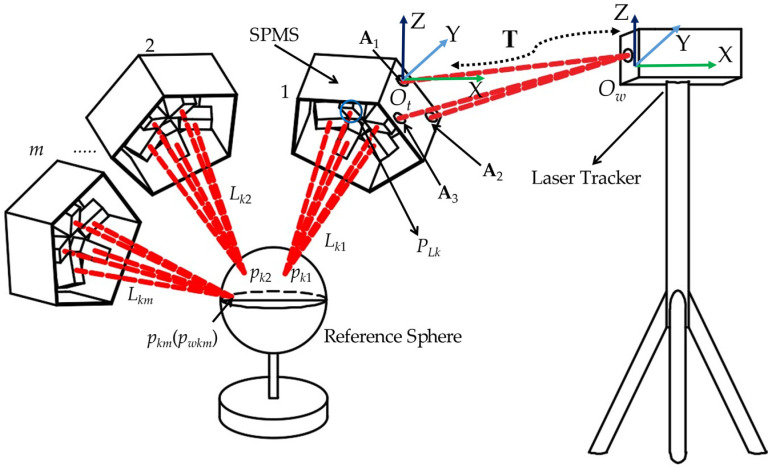
The data acquisition procedure of laser beams based on reference sphere constraint.

As shown in Figure 3, *p_k_* is the flare on the reference sphere of sensor *k*; **F***_k_* is the coordinate of *p_k_*; *O_t_* is the measurement coordinate system; *O_w_* is the world system attached with the laser tracker; **P***_Lk_* = [*x_Lk_*, *y_Lk_*, *z_Lk_*]*^T^*; **γ***_k_* = [*γ_kx_*, *γ_ky_*, *γ_kz_*]*^T^* and *γ_kx_*^2^ + *γ_ky_*^2^ + *γ_kz_*^2^ = 1; and **T** is the homogeneous transformation matrix between *O_t_* and *O_w_*; *m* is the number of pose changes of SPMS. Three points A_1_, A_2,_ and A_3_ are selected on the surface of the bracket of SPMS, with which *O_t_* is established by Schmidt orthogonalization and normalization. Then, according to (2), the nonlinear least squares problem is established by orthogonal decomposition and homogeneous transformation. Finally, the position and orientation parameters of the laser beam in *O_t_* will be obtained by solving the nonlinear least square problem.

### 3.2. Establishment of the Measurement Coordinate System

To determine the origin and the axis direction of *O_t_*, three points **A**_1_, **A**_2,_ and **A**_3,_ on the surface of SPMS are selected, which will be measured by the laser tracker. **A**_1_ is the origin of *O_t_*. According to the calculation of **A**_1_, **A**_2,_ and **A**_3_, we can obtain two vectors, **b** and **c**, as shown in (4)

(4) $$\begin{array}{l} b=A_{2}-A_{1}\\ c=A_{3}-A_{1} \end{array}$$

The orthogonal vector of **b** and **c** is:

(5) d=b×c

By Schmidt orthogonalization of vectors **b**, **c,** and **d**, the corresponding three mutually orthogonal vectors **β**_1_, **β**_2,_ and **β**_3_ can be calculated by:

(6) $$\begin{array}{l} \beta_{1}=b\\ \beta_{2}=c-\frac{\langle c,\beta_{1}\rangle }{\langle \beta_{1},\beta_{1}\rangle }\beta_{1}\\ \beta_{3}=d-\frac{\langle d,\beta_{1}\rangle }{\langle \beta_{1},\beta_{1}\rangle }\beta_{1}-\frac{\langle d,\beta_{2}\rangle }{\langle \beta_{2},\beta_{2}\rangle }\beta_{2} \end{array}$$

By normalization of vectors **β**_1_, **β**_2,_ and **β**_3_, the corresponding orthogonal unit vectors **α**_1_, **α**_2,_ and **α**_3_ can be obtained. Taking **A**_1_ as the origin, **α**_1_, **α**_2,_ and **α**_3_ as *x* axis, *y* axis, and *z* axis, the measurement coordinate system *O_t_* can be established. Since the coordinates of **A**_1_, **A**_2,_ and **A**_3_ are measured by the laser tracker in its measurement coordinate system *O_w_*, we can obtain the homogeneous transformation matrix **T**:

(7) $$T=\left[\begin{array}{cccc} \alpha_{11} & \alpha_{21} & \alpha_{31} & A_{1x}\\ \alpha_{12} & \alpha_{22} & \alpha_{32} & A_{1y}\\ \alpha_{13} & \alpha_{23} & \alpha_{33} & A_{1z}\\ 0 & 0 & 0 & 1 \end{array}\right]$$

where *α_ij_* is the component of **α***_i_* (*I* = 1, 2, 3; *j* = 1, 2, 3); *A*_1*x*_, *A*_1*y,*_ and *A*_1*z*_ are the coordinates of **A**_1_ in *O_w_*.

### 3.3. Identification Modeling of Position and Orientation for Laser Beams

As shown in Figure 3, SPMS can be carried by a robot or other machines to move and be measured in different positions and orientations. The reference sphere is fixed, and the coordinates of the center of the reference sphere, which is fixed, are **P***_Bw_* = [*x_Bw_*, *y_Bw_*, *z_Bw_*]*^T^* in *O_w_*.

The coordinate system *O_t_* is moving with SPMS in the calibration process. The coordinates in *O_t_* will be transformed into *O_w_* for consistency. In *O_t_*, the coordinates of the flare on the reference sphere of sensor *k*, i.e., **F***_k_* in *O_t,_* can be calculated:

(8) $$F_{k}=P_{L}{}_{k}+\gamma_{k}L_{k}$$

The homogeneous coordinates of **F***_k_* are as shown in (9), which will be used in the next homogeneous transformation.

(9) $$F_{k4\times 1}=\left[\begin{array}{c} F_{k}\\ 1 \end{array}\right]$$

By multiplying the transformation matrix **T**, the homogeneous coordinates **F***_k_*_4×1_ in *O_w_* can be obtained by (10).

(10) $$F_{wk4\times 1}=T\cdot F_{k4\times 1}$$

By substituting **P***_Lk_* = [*x_Lk_*, *y_Lk_*, *z_Lk_*]*^T^* and **γ***_k_* = [*γ_kx_*, *γ_ky_*, *γ_kz_*]*^T^* into the equation, the coordinates of **F***_k_* in *O_w_* can be derived by (8)–(10).

(11) $$\left[\begin{array}{c} \begin{array}{c} x_{wk}\\ y_{wk}\\ z_{wk} \end{array}\\ 1 \end{array}\right]=T\cdot \left[\begin{array}{c} \begin{array}{c} x_{Lk}+\gamma_{kx}L_{k}\\ y_{Lk}+\gamma_{ky}L_{k}\\ z_{Lk}+\gamma_{kz}L_{k} \end{array}\\ 1 \end{array}\right]$$

where *x_wk_*, *y_wk_*, and *z_wk_* are the coordinates of **F***_k_* in *O_w_*, respectively. Because **F***_k_* is a point on the surface of the reference sphere, (12) can be obtained according to (2) and (11).

(12) $${\left(x_{wk}-x_{Bw}\right)}^{2}+{\left(y_{wk}-y_{Bw}\right)}^{2}+{\left(z_{wk}-z_{Bw}\right)}^{2}=R^{2}$$

To obtain the least squares solution of *x_Lk_*, *y_Lk_*, *z_Lk_*, *γ_kx_*, *γ_ky,_* and *γ_kz_* of (11), (11) and (12) are rewritten as the function of *d_k_*, which is the distance between **F***_k_* and **P***_Bw_*:

(13) $$d_{k}=f\left(x_{Lk},y_{Lk},z_{Lk},\gamma_{kx},\gamma_{ky},\gamma_{kz},x_{Bw},y_{Bw},z_{Bw}\right)$$

At *m* positions and orientations, SPMS can be measured *m* times, by which *m* groups of data, including *L_ki_* and **A**_1*i*_, **A**_2*i*_, **A**_3*i*_ (*i* = 1, 2, …, *m*), can be obtained. With these data, *m* equations can be obtained according to (13). The solution of these equations can be converted to a least square problem, as shown in (14).

(14) $$\begin{array}{l} \Delta \eta =[\Delta P_{Lk},\Delta \gamma_{k},\Delta P_{Bw}]\\ \min\;f(\Delta \eta )=\sum_{i=1}^{m}\Phi (\Delta \eta )={\sum_{i=1}^{m}\left\|d_{ki}-R\right\|}^{2} \end{array}$$

There are *K* laser range sensors in SPMS, and the position and orientation of the laser beam of each sensor have six parameters. Thus, (14) has 6*K* + 3 unknown variables, including 3 for the coordinates of the center of the reference sphere. Thereby, to ensure that (14) has definite solutions, *m* must be greater than or equal to 6*K* + 3.

## 4. Parameter Identification of Position and Orientation of Laser Beams

Solving (14), we can obtain the position and orientation of the laser beam of each sensor, as well as the coordinates of the reference spherical center. The Levenberg–Marquardt (LM) algorithm is a common method for solving nonlinear least squares problems with the advantages of fast convergence and strong robustness. However, (14) is a high-dimensional and strong nonlinear system of equations. When LM was used to solve (14), the results depended heavily on the initial value. Particle swarm optimization (PSO) is a swarm intelligence optimization algorithm that seeks the optimal solution through cooperation and information sharing among particles in the population. Therefore, PSO is suitable for solving multivariable and strongly nonlinear problems, but its calculation results have a certain extent of randomness. We present a PSO-LM hybrid algorithm to solve (14), where PSO is used to obtain the initial solution, and LM is used for the final solution.

### 4.1. Initial Parameter Identification Based on PSO

To identify the 6*K* + 3 parameters in (14), the particle population size *t* of the PSO is set as 5000. Then, the dimensions of position vector **X***_n_*_−1_*^i^* and velocity vector **V***_n_*_−1_*^i^* of the *i*th particle are all 6*K* + 3 in the *n* − 1th iteration, and the iterations of **X***_n_*_−1_*^i^* and **V***_n_*_−1_*^i^* are:

(15) $$\begin{array}{l} V_{n}^{i}=wV_{n-1}^{i}+c_{1}r_{1}(P_{{}_{best}n-1}^{i\;}-X_{n-1}^{i})+c_{2}r_{2}(G_{{}_{best}n-1}^{}-X_{n-1}^{i})\\ X_{n}^{i}=X_{n-1}^{i}+V_{n}^{i} \end{array}$$

where **P***^i^_best n_*_−1_ is the best solution of the *i*th particle in the *n* − 1th iteration; **G***_best n_*_−1_ is the best solution of all the particles in the *n* − 1th iteration; *c*_1_ and *c*_2_ are the learning factors; generally, *c*_1_ = *c*_2_ = 2 [27,28]; *r*_1_ and *r*_2_ are random numbers with a value range of [0, 1]; and *w_n_* is the inertial factor, which can be calculated by (16).

(16) $$w_{n}=w_{\max}-\frac{(w_{\max}-w_{\min})n^{2}}{g_{n}^{2}}$$

where *w*_max_ and *w*_min_ are the upper and lower bounds of the inertia factor. Generally, *w*_max_ = 0.9, *w*_min_ = 0.4, and *g_n_* are the maximum number of iterations.

### 4.2. Final Parameter Identification Based on LM

According to the basic principle of the LM algorithm, the *n* + 1th iteration equation in solving (14) is:

(17) $$\delta_{n+1}=\delta_{n}-{\left(J_{}^{T}J_{}+\lambda_{n}I\right)}^{-1}J_{n}^{T}\varepsilon_{n}$$

where **δ***_n_* is a (6*K* + 3) × 1 vector; **J** is a (*K* × *m*) × (6*K* + 3) Jacobian matrix; **I** is a (6*l* + 3) × (6*l* + 3) identity matrix; **ε***_n_* is a (*K* × *m*) × 1 fitting error vector of the reference sphere; and *λ_n_* is the damping factor. Let:

(18) $$J_{P_{Lkn}}=\left[\begin{array}{ccc} \frac{\partial d_{k1n}}{\partial x_{Lk}} & \frac{\partial d_{k1n}}{\partial y_{Lk}} & \frac{\partial d_{k1n}}{\partial z_{kL}}\\ & ... & \\ \frac{\partial d_{kmn}}{\partial x_{Lk}} & \frac{\partial d_{kmn}}{\partial y_{Lk}} & \frac{\partial d_{kmn}}{\partial z_{Lk}} \end{array}\right]$$

(19) $$J_{\gamma_{kn}}=\left[\begin{array}{ccc} \frac{\partial d_{k1n}}{\partial \gamma_{xk}} & \frac{\partial d_{k1n}}{\partial \gamma_{yk}} & \frac{\partial d_{k1n}}{\partial \gamma_{zk}}\\ & ... & \\ \frac{\partial d_{kmn}}{\partial \gamma_{xk}} & \frac{\partial d_{kmn}}{\partial \gamma_{yk}} & \frac{\partial d_{kmn}}{\partial \gamma_{zk}} \end{array}\right]$$

(20) $$J_{P_{Bwkn}}=\left[\begin{array}{ccc} \frac{\partial d_{k1n}}{\partial x_{Bw}} & \frac{\partial d_{k1n}}{\partial y_{Bw}} & \frac{\partial d_{k1n}}{\partial z_{Bw}}\\ & ... & \\ \frac{\partial d_{kmn}}{\partial x_{Bw}} & \frac{\partial d_{kmn}}{\partial y_{Bw}} & \frac{\partial d_{kmn}}{\partial z_{Bw}} \end{array}\right]$$

where *d_kmn_* is the distance between **p***_k_* and **P***_Bw_* in the *n*th iteration; **J***_PLkn_*, **J***_γkn,_* and **J***_PBkn_* are the first-order partial derivative matrices of *d_kmn_* for each component of **P***_Lk_*, **γ***_k,_* and **P***_wBk_*; *m* is the number of acquisition data; and *k* is the number of the laser range sensor. Then, the Jacobian matrix **J** of laser beam position parameters will be:

(21) $$J=\left[\begin{array}{ccccccc} J_{PL1n} & & & J_{\gamma_{1n}} & & & J_{P_{Bw1n}}\\ & ... & & & ... & & ...\\ & & J_{PLkn} & & & J_{\gamma_{kn}} & J_{P_{Bw\mathrm{kn}}} \end{array}\right]$$

The initial value of the damping factor *λ*_0_ is:

(22) $$\lambda_{0}=\tau \cdot \max\left\{\max\left(J^{T}J\right)\right\}$$

where *τ* = 10^−8^, its value is generally obtained from experience and adjusted according to actual calculation results. The iteration value of the damping factor *λ_n_* is determined according to the scale factor *ρ_n_*:

(23) $$\rho_{n}=\frac{X_{n}^{T}(\lambda_{n-1}X_{n}-J^{T}\varepsilon_{n})}{2}$$

The iteration of the damping factor *λ_n_* is:

(24) $$\left\{\begin{array}{l} \lambda_{n+1}=\lambda_{n}\cdot \max\{\frac{1}{3},1-(2\rho_{n}-1{)}^{3}\},\rho_{n}\geq 0\\ \lambda_{n+1}=\lambda_{n}\kappa_{n},\kappa_{n+1}=2\kappa_{n},\rho_{n}<0 \end{array}\right.$$

## 5. Results of Experiments and Discussion

Three experiments were conducted to verify the proposed measurement system and parameter identification method, including parameter identification of the position and orientation of laser beams, verification of measuring accuracy of SPMS, and kinematic calibration of industrial robots in this section. During the experiments, the ambient temperature is about 20 °C, and the change is small. When the laser range sensor is not preheated, its measurement accuracy is unstable [29]. Therefore, the laser range sensor was preheated for 30 min before the experiment. After the completion of preheating, the experimental data will be collected and processed.

### 5.1. Experiments for Identification of Laser Beams

**Figure 4:**
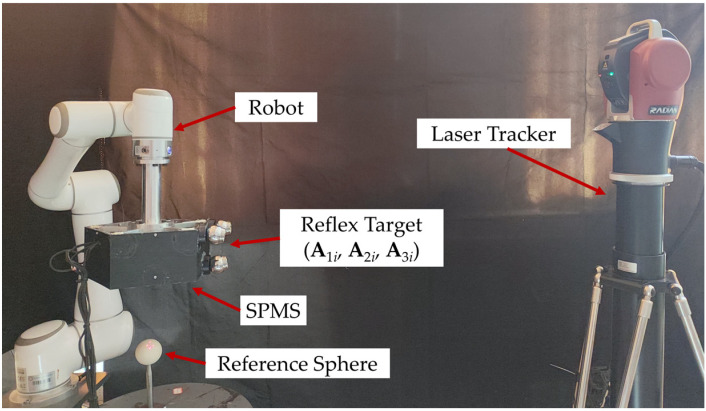
The platform for parameter identification and measuring accuracy.

The experimental platform for the calibration of laser beams and verification of measuring accuracy consists of SPMS, a laser tracker, an EC66 robot, and a reference sphere with a diameter of 50.8143 mm, as shown in Figure 4. The EC66 robot is only used as an actuator carrying SPMS to change position and orientation.

**Figure 5:**
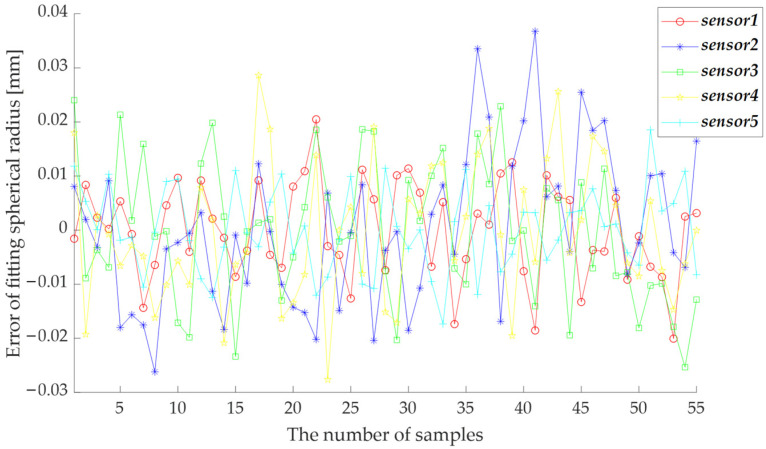
The spherical fitting error of each group of data.

SPMS was carried by the EC66 robot to 55 different positions and orientations, and 55 groups of *L_k_*, **X**_1_, **X**_2,_ and **X**_3_ were acquired. With these acquired data, the position and orientation parameters were obtained by the proposed identification method based on PSO-LM, as shown in Table 2. The spherical fitting error of each group of data is illustrated in Figure 5, which shows that nearly all the errors are smaller than 0.03 mm. The measuring accuracy, which can be indicated by the maximum measuring error required by the calibration of industrial robots, is 0.2 mm. Thus, the measuring accuracy of SPMS can meet the requirement. The measuring accuracy will be further verified in the next section. After calibration, the components of the position and orientation of **P***_L__k_* = [*x_Lk_*, *y_Lk_*, *z_Lk_*]*^T^* and **γ***_k_* = [*γ_kx_*, *γ_ky_*, *γ_kz_*]*^T^* are shown in Table 2.

### 5.2. Verification of the Measuring Accuracy

**Figure 6:**
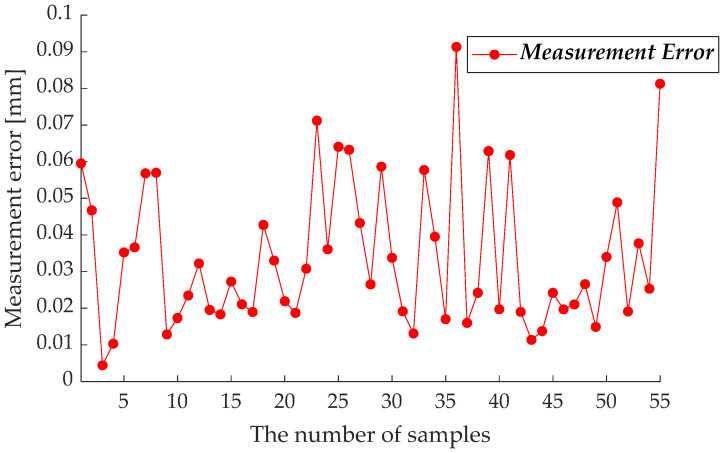
The measuring errors of SPMS.

To verify the measuring accuracy of SPMS, an API R-20 Radian laser tracker is used as the reference to calculate the measuring error of SPMS, as shown in Figure 4. The reference sphere was measured 55 times using SPMS from different directions. With the measured data **P**_B_ and **P***_Bw_* can be obtained by (3) and (13), respectively. The difference between **P**_B_ and **P***_Bw_* is taken as the measuring error. The measuring errors of SPMS in 55 samples are shown in Figure 6, and the statistical information is shown in Table 3. The maximum error and average error of SPMS are 0.091 mm and 0.034 mm, respectively, which are precision enough to be applied in the calibration of industrial robots. The measurement error of SPMS mainly comes from the linearity error of laser range sensors according to the analysis and test.

### 5.3. Experiments of Calibration for an Industrial Robot

**Figure 7:**
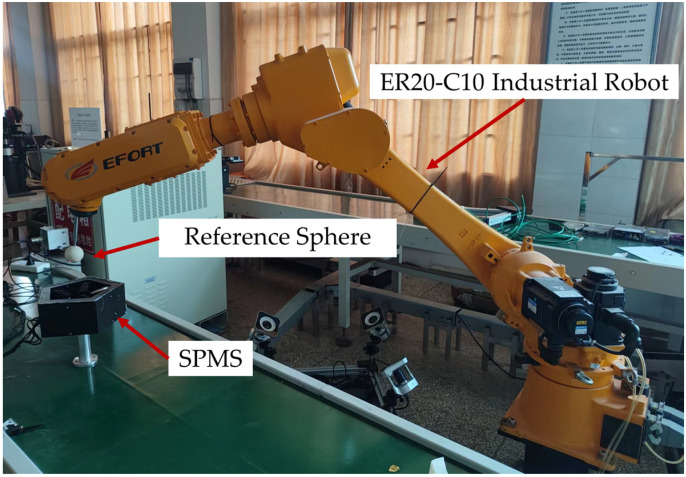
The experiment platform for kinematic calibration of robots.

To further verify the effectiveness of SPMS in the calibration of industrial robots, the SPMS was used to calibrate an ER20-C10 6-DoF industrial robot. The kinematic model was established according to the modified Denavit–Hartenberg (MD-H). The position of the robot’s end was measured with SPMS. With the measured data, kinematic parameters and the transformation parameter from the base of the robot to the measuring coordinate of SPMS, as well as the parameters from the flange to the reference sphere, were identified with the method in our previous publication [30]. As shown in Figure 7, the experimental platform for kinematic calibration of robots consists of SPMS, ER20-C10 robot, and a reference sphere with a diameter of 50.8143 mm.

The nominal kinematic parameters of the ER20-C10 robot before calibration are shown in Table 4.

With the acquired data by SPMS, the kinematic parameters of the ER20-C10 industrial robot are identified, as shown in Table 5.

**Figure 8:**
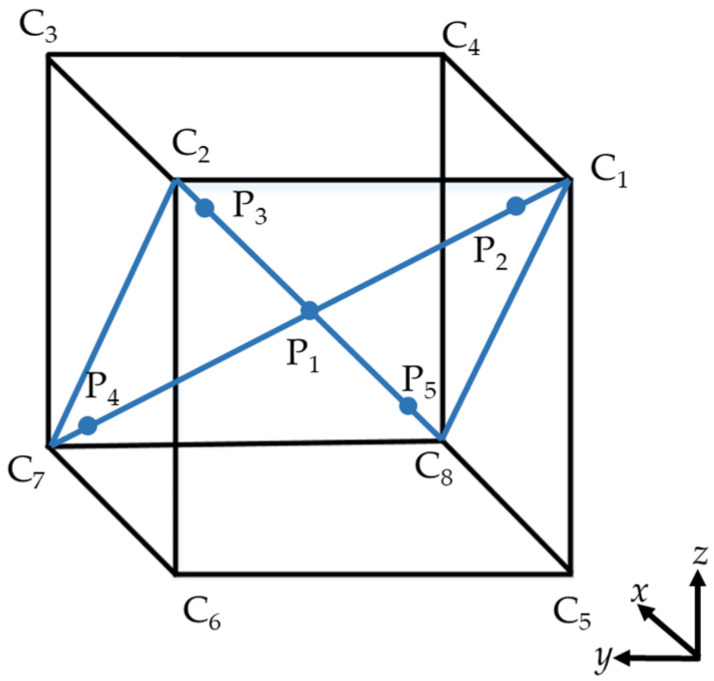
The test cube of the ER20-C10 industrial robot.

The identified kinematic parameters are compensated to the controller of the ER20-C10 industrial robot. The positioning accuracy of the industrial robot is tested according to ISO 9283:1998 [31], where a cube of 400 mm × 400 mm × 400 mm was created in the working space of the ER20-C10 industrial robot, shown in Figure 8. C_1_~C_8_ are the eight vertices of the cube. Five testing points P_1_~P_5_ on the diagonal plane C_1_-C_2_-C_7_-C_8_ of the cube are determined, among which P_1_ is the center of the cube, P_2_~P_5_ are on the two diagonals of C_1_-C_2_-C_7_-C_8_ and being away from the vertices (10 ± 2)% of the diagonal length. After the cube was determined, reflex targets were installed at the end of the ER20-C10 robot for measurement with the laser tracker. According to ISO 9283:1998, cycle measurement 30 times should be conducted, and the average errors of P1~P5 can be obtained to evaluate the absolute positioning accuracy of the industrial robot. In the experiments, the absolute positioning (AP) was tested by the laser tracker before and after calibration, respectively. The results are shown in Table 6, which indicates that the maximum error, average error, and RMS are reduced from 1.462 mm, 1.056 mm, and 0.829 mm to 0.712 mm, 0.461 mm, and 0.388 mm, with the reduction percentage of 51.3%, 56.3%, and 53.2%, respectively. In another study by our team [30], a Radian laser tracker was used to calibrate and test an ER20-C10 industrial robot, and the maximum error, average error, and std of the robot after calibration were 0.637 mm, 0.460 mm, and 0.184 mm. Although there is still a certain gap between the calibration effect of SPMS and that of the laser tracker, the kinematic calibration of the industrial robot based on SPMS is effective and can significantly improve the absolute accuracy of the industrial robot.

## 6. Conclusions

A spatial position measurement system (SPMS) based on redundant laser range sensors is proposed to solve industrial robots’ measurement and calibration problems in narrow industrial sites. The main work of this paper is as follows:

▪ The conversion model is established from the distance information acquired by the laser range sensor to position information through the standard spherical constraint;
▪ The parameters of position and orientation of laser range sensors in the model are identified by a hybrid algorithm of PSO-LM to solve the high-dimensional and strong nonlinear problem of the model;
▪ Experiments were carried out to verify the conversion model, the identification method, the measuring accuracy of SPMS, and the effectiveness of industrial robot calibration. The results of the experiments show that the maximum and average error of SPMS is 0.091 mm and 0.034 mm, respectively; after calibration, the maximum error, average error, and RMS of the industrial robot are reduced from 1.462 mm, 1.056 mm, and 0.829 mm to 0.712 mm, 0.461 mm, and 0.388 mm, with the reduction percentage of 51.3%, 56.3%, and 53.2%, respectively.

Therefore, the proposed measurement device based on redundant laser range sensors is precise enough and can achieve suitable performance for industrial robot calibration.

Although many factors have been considered in the research work of this paper, there are still some problems that need to be considered or improved in future work: (1) Research on temperature compensation should be carried out to achieve more stable measurement accuracy; (2) The influence of measurement configuration on industrial robot calibration is not considered. The measurement configuration has a certain influence on the calibration results. To further improve the effect of SPMS on the kinematic calibration of industrial robots, optimization of the measurement configuration in the kinematic calibration of industrial robots based on the observability index should be studied.

## Figures and Tables

**Table 1:** The performance parameters of the range sensor.

Standard Distance	Measurement Range	Linearity	Repeatability	Temperature Characteristics	Ambient Temperature ^2^
100 mm	75 mm to 130 mm	±0.15% of F.S. ^1^	10 μm	0.06% of F.S./°C	−10 to +50 °C

^1^ F.S. denotes full scale. ^2^ No condensation or freezing.

**Table 2:** The identified position and orientation parameters of laser beams.

*k*	*x_Lk_* (mm)	*y_Lk_* (mm)	*z_Lk_* (mm)	*γ_xk_* (rad)	*γ_yk_* (rad)	*γ_zk_* (rad)
1	74.420	67.247	97.126	−0.104	0.976	0.189
2	11.612	69.074	146.755	0.210	0.975	−0.069
3	92.918	67.986	142.254	−0.190	0.981	−0.031
4	52.951	69.811	175.249	0.018	0.981	−0.194
5	25.346	70.181	97.548	0.143	0.982	0.124

**Table 3:** The statistics information of the measuring errors.

Index	Maximum Error	Average Error	RMS
Value (mm)	0.091	0.034	0.039

**Table 4:** The kinematic parameters of the ER20-C10 robot before calibration.

No. of Joints	*α_i_*_−1_ (rad)	*a_i_*_−1_ (mm)	*d_i_* (mm)	*δθ_i_* (rad)
1	0	0.000	504.000	*0*
2	−pi/2	166.605	0.000	0
3	0	−782.270	0.000	*0*
4	−pi/2	138.826	761.350	*0*
5	pi/2	0.00	0.000	*0*

**Table 5:** The kinematic parameters of the ER20-C10 robot after calibration.

No. of Joints	*α_i_*_-1_ (rad)	*a_i_*_-1_ (mm)	*d_i_* (mm)	*δθ_i_* (rad)
1	0	0.000	504.000	0
2	−pi/2	167.648	0.000	1.39 × 10^−2^
3	0	−780.753	0.000	1.92 × 10^−4^
4	−pi/2	139.918	759.330	3.84 × 10^−4^
5	pi/2	0.00	0.000	6.63 × 10^−4^

**Table 6:** The nominal kinematic parameters of the ER20-C10 robot after calibration.

Position	AP (mm) Before Calibration	AP (mm) After Calibration
P1	0.336	0.167
P2	1.462	0.712
P3	0.997	0.496
P4	1.454	0.620
P5	1.030	0.311

## Data Availability

All data will be made available on request to the correspondent author’s e-mail with appropriate justification.
